# A Responsible AI Readiness Framework for Digital Self-Management Platforms: Illustrative Application to a Rule-Based GERD Platform

**DOI:** 10.3390/healthcare14183061

**Published:** 2026-09-17

**Authors:** Sun-Young Kang, Joosung Lee, Jeong-An Gim

**Affiliations:** 1 Institute for Molecular Metabolism Innovation, Soonchunhyang University, Asan 31538, Republic of Korea; rloveush@sch.ac.kr; 2 Enterprise School, Soonchunhyang University, Asan 31538, Republic of Korea; 3 Department of Medical Science, Soonchunhyang University, Asan 31538, Republic of Korea

**Keywords:** responsible artificial intelligence, digital health, clinical governance, implementation readiness, digital therapeutics, self-management, gastroesophageal reflux disease, human oversight

## Abstract

**Highlights:**

**What are the main findings?**

- A pre-adoption framework combines an AI necessity gate with eight domains spanning clinical evidence, safety, data governance, transparency, equity, implementation capacity, regulatory–economic planning, and environmental stewardship.
- The content audit classified 18 of 20 fixed mission statements as requiring revision and 2 as retainable, and a non-version-linked interface design record contained legacy symptom-improvement and personalization wording that exceeded the intended self-management claim boundary.

**What are the implications of the main findings?**

- AI should be added only when it provides prespecified, clinically meaningful value beyond a fixed rule-based comparator and can be governed throughout the lifecycle.
- Retaining or improving a simpler rule-based system is a responsible outcome when AI would add burden without adequate benefit, evidence, or implementation capacity.

**Abstract:**

**Background/Objectives**: Artificial intelligence (AI) is increasingly proposed for digital self-management, yet early-stage platforms may not be clinically or organizationally ready for AI-enabled functions. Most responsible-AI guidance begins after a model has been proposed and gives limited attention to whether AI should be introduced at all. This Perspective proposes a Responsible AI Readiness Framework for digital self-management platforms. **Methods**: The framework was developed through a targeted integrative synthesis, concept extraction, domain consolidation, and comparison with established digital-health and AI frameworks. It was applied qualitatively, without scoring, to Sokcare, a rule-based mobile platform for gastroesophageal reflux symptoms. No participant-level data were analyzed. **Conceptual Findings**: The framework begins with an AI necessity and proportionality screen and then examines eight readiness domains. In the Sokcare audit, 18 of 20 fixed mission statements were classified as Revise and 2 as Retain; none were classified as Remove. A non-version-linked interface design record also contained legacy symptom-improvement and personalization wording that exceeded the intended self-management claim boundary. The case otherwise showed potential foundations in interpretability, user agency, and modular architecture, alongside gaps in clinical validation, safety escalation, cybersecurity, accessibility, implementation, and lifecycle monitoring. **Conclusions**: The framework may support structured pre-adoption AI decisions, but its transferability and decision consistency require testing across independent platforms, clinical areas, and regulatory settings.

## 1. Introduction

Digital self-management platforms can extend education, symptom tracking, reminders, and behavior support beyond clinical encounters. Their value depends on bounded claims, safety escalation, equitable access, workflow fit, ownership, and lifecycle capacity—not technical availability alone [1,2].

AI may support pattern recognition and adaptive intervention, but can also create bias, opacity, privacy, cybersecurity, and organizational burdens. Responsible health-AI guidance therefore emphasizes safety, transparency, accountability, equity, and lifecycle risk management [3,4,5,6,7].

Most responsible-AI guidance begins after an AI model or AI-enabled device has been proposed. A prior healthcare question receives less attention: should an early digital self-management platform introduce AI at all? Transparent deterministic rules may be safer, easier to audit, less data-dependent, and easier to integrate when the decision problem is stable and AI’s incremental clinical or operational value has not been established. Environmental proportionality also remains relevant because computationally intensive systems can add resource use without commensurate benefit [8,9,10]. Responsible AI readiness should therefore include both the capacity to adopt AI safely and the discipline to retain a simpler system when AI is unnecessary, ungovernable, or disproportionate.

The same issue arises in the transition from wellness-oriented software to digital therapeutics (DTx). Software as a Medical Device (SaMD) is defined by its intended medical purpose rather than by whether it is delivered by prescription [11]. Products that claim to prevent, manage, or treat a condition require an evidence and quality pathway proportionate to their intended use, including analytical and clinical evaluation, risk management, cybersecurity, maintenance, and post-market monitoring [11,12,13]. The DTx Real-World Evidence Framework further emphasizes iterative design, testing, deployment, and monitoring throughout the product lifecycle [14]. In Korea, the regulatory and reimbursement environment for DTx is developing, but evidence generation, clinical integration, adoption, and market access remain major challenges [15]. Thus, an early mobile platform should not be labeled a DTx merely because it delivers health content; DTx positioning follows from a defined medical purpose, adequate evidence, and a feasible implementation pathway.

GERD is heterogeneous, and similar symptoms can reflect different conditions and care pathways [16,17,18]. A consumer platform may support observation and low-risk behavior change, but should not infer diagnosis, modify medication, or present general self-management as proven treatment [19,20,21,22,23,24,25,26,27,28,29].

GERD apps show that user-centered design and symptom diaries are feasible, yet evidence provenance, professional involvement, safety, privacy, and quality vary [30,31,32,33,34,35]. Little work addresses how a transparent rule-based platform should be assessed before AI changes its claim, data, and governance burden.

This Perspective addresses two questions: (1) what evidence and governance conditions should be considered before AI is added to a rule-based digital self-management platform; and (2) how can these conditions be illustrated using a versioned, author-conducted case? The framework is intended primarily for AI functions that may alter health-related recommendations, risk classification, or behavior-support decisions. Its application should be proportionate to intended use and risk.

This Perspective proposes a Responsible AI Readiness Framework for digital self-management platforms and illustrates it with Sokcare, a rule-based reflux-symptom self-management platform. The framework starts with an AI necessity and proportionality gate, followed by eight readiness domains. The worked example identifies documented assets and unresolved gaps. A staged pathway then connects bounded self-management software to an evaluated digital health intervention and regulated DTx; AI-assisted DTx is considered only when justified. This contribution is not an evaluation of clinical efficacy or AI performance.

## 2. Clinical and Implementation Context

### 2.1. Healthcare Implementation and System Readiness

Digital self-management contributes to healthcare only when its purpose, safety boundary, user pathway, and organizational ownership are explicit. Readiness therefore concerns governance, interoperability, workforce capacity, equitable access, and feasible links to information, referral, and human support [1].

### 2.2. From Digital Self-Management to DTx

A self-management application may provide education, monitoring, prompts, and behavior planning without claiming diagnosis or treatment. A DTx instead requires evidence and controls proportionate to its intended medical purpose, including clinical association, technical performance, clinical validation, safety, and quality management [11,13].

A wellness-first strategy must not bypass appropriate evidence or regulation. Clinical claims should advance only after the intervention, risk controls, workflows, and evaluation plan are fixed; evidence generation and monitoring then continue across the lifecycle [14,15,36,37].

Accordingly, this paper distinguishes a rule-based self-management platform, an evaluated digital health intervention, regulated DTx, and AI-enabled DTx. The pathway should remain proportionate to intended function and risk. Low-risk administrative, reminder, or engagement functions that do not alter clinical recommendations, risk classification, or behavior-support decisions do not automatically require a DTx pathway.

### 2.3. Why Readiness Must Precede AI Adoption

AI readiness is not only a matter of data, infrastructure, or model skill. A platform may still be unready when its purpose, outcomes, safety pathway, target population, professional workflow, or governance ownership is unclear; a clinically bounded rule system may then be preferable.

Gate 0 therefore asks whether AI offers meaningful incremental value over explicit rules and whether representative data, valid labels, human oversight, lifecycle monitoring, and proportionate resources are available. A decision to retain the simpler system is a responsible outcome, not a failure of innovation.

## 3. Framework Development and Case-Application Approach

### 3.1. Targeted Integrative Synthesis

The framework was developed through a targeted integrative synthesis, not a systematic review. Searches through 4 August 2026 covered PubMed/MEDLINE and the official websites of WHO, the United Nations, NIST, IMDRF, and the Korean Ministry of Food and Drug Safety, supplemented by backward citation checking. Three PubMed/MEDLINE blocks retrieved 423, 769, and 56 records (1248 total; 1197 unique after deduplication), of which 7 were retained directly; 9 authoritative documents and 40 sources identified through backward citation checking or targeted supplementation gave a final set of 56 sources for framework construction and case-boundary mapping. Complete search blocks, eligibility principles, extraction fields, and selection details are provided in Supplementary Methods S1 and Table S2.

Sources were organized across digital-health implementation, responsible health AI, environmental proportionality, medical-device software and DTx regulation, digital-intervention development and reporting, and GERD clinical and digital-health evidence. Table 1 summarizes each source family and its contribution to the framework.

Eligible sources informed a pre-AI decision, evidence requirement, or case boundary. Because the aim was conceptual construction rather than exhaustive effect estimation, no pooled estimates or PRISMA flow were produced.

For each source, we recorded the decision question, failure mode, minimum evidence or governance artifact, responsible actor, lifecycle stage, and implementation dimension. Eight domains were retained when omission could independently block safe progression or required a distinct evidence package or owner; Gate 0 was retained because it precedes, and may end, AI development. The complete derivation and source-to-element audit trail are in Supplementary Table S3A,B.

S.-Y.K. conducted initial source identification and extraction. During revision, J.L. independently reviewed Gate 0, domain assignments, and primary mappings; J.-A.G. independently reviewed GERD evidence, safety boundaries, and all 20 item–mission mappings. Disagreements were resolved by consensus, using the more conservative interpretation when uncertainty remained.

### 3.2. Case Application

Sokcare was selected as an illustrative case because it is a functioning rule-based self-management platform at the point where AI, prospective clinical evaluation, or DTx positioning could be considered but have not been justified. The fixed technical artifacts comprised the Flutter source snapshot (version 1.0.5+14), interface screens, 20 lifestyle items, 20 linked missions, reminder and reporting logic, SharedPreferences local storage, Supabase event storage, and a web-based administrative interface. No participant-level data, actual user logs, or outcomes from the earlier service pilot were analyzed in this paper.

The case was assessed qualitatively and without numerical scoring. For each domain, we identified (a) foundations supported by fixed artifacts and development documentation; (b) readiness gaps; and (c) the activity required before progression. Eight categories of formative consultation records—development strategy, epidemiology and research design, economic considerations, overseas certification, two gastroenterology perspectives, family medicine, and psychiatry—were used only to explain development priorities. They were not treated as a quantitative validation.

Because S.-Y.K. developed the Sokcare platform and is named as an inventor on the related patent application, the Sokcare assessment was not independent. J.L. and J.-A.G. performed a secondary review of the framework mapping and the clinical and safety interpretation, respectively; however, both are coauthors. The case should therefore be interpreted as an author-generated worked example rather than an external validation, an independent certification, or a platform audit. An earlier service-development pilot involved 30 users; however, no pilot-user data, questionnaire responses, usage logs, or outcomes were accessed or analyzed in the present Perspective. The pilot was conducted as a service-development activity without a prospective IRB determination, and any future research use of those historical data would require prospective institutional review.

### 3.3. Interpretation Boundary and Ethics

The framework is a preliminary conceptual framework and should not be interpreted as a validated assessment instrument. It has not undergone Delphi consensus, psychometric validation, external validation, or inter-rater reliability testing. The Sokcare application is an author-conducted worked example intended to illustrate how the proposed framework may identify documented foundations and unresolved gaps; it should not be interpreted as independent certification, clinical validation, regulatory approval, or a benchmark score.

This study analyzes software artifacts and non-research development documents. It includes no new human participants, identifiable personal information, or analysis of service-user data. Institutional review board approval and informed consent were therefore not applicable to the analyses reported here. Any future expert rating, usability study, clinical investigation, or secondary analysis of user data should receive prospective institutional determination and appropriate consent procedures.

### 3.4. Use of Generative Artificial Intelligence

During manuscript preparation and revision between July and September 2026, the authors used ChatGPT (GPT-5.6, OpenAI) under a Pro subscription to assist with language editing, manuscript organization, literature-search planning, and draft-language generation. The authors reran the literature searches, checked each citation against the original publication or official source, and verified all scientific claims, framework content, figure labels, and case interpretation. Full details are provided in the GenAI Disclosure statement.

Table 2 compares the proposed framework with established digital-health and AI frameworks. The comparisons describe each source’s stated scope and intended use; they are not quality ratings. The proposed framework integrates rather than replaces prior work. Its narrower contribution is to operationalize a pre-adoption decision around a fixed rule-based comparator while bringing clinical, implementation, regulatory–economic, equity, and environmental readiness into one workflow.

## 4. Proposed Responsible AI Readiness Framework

**Figure 1:**
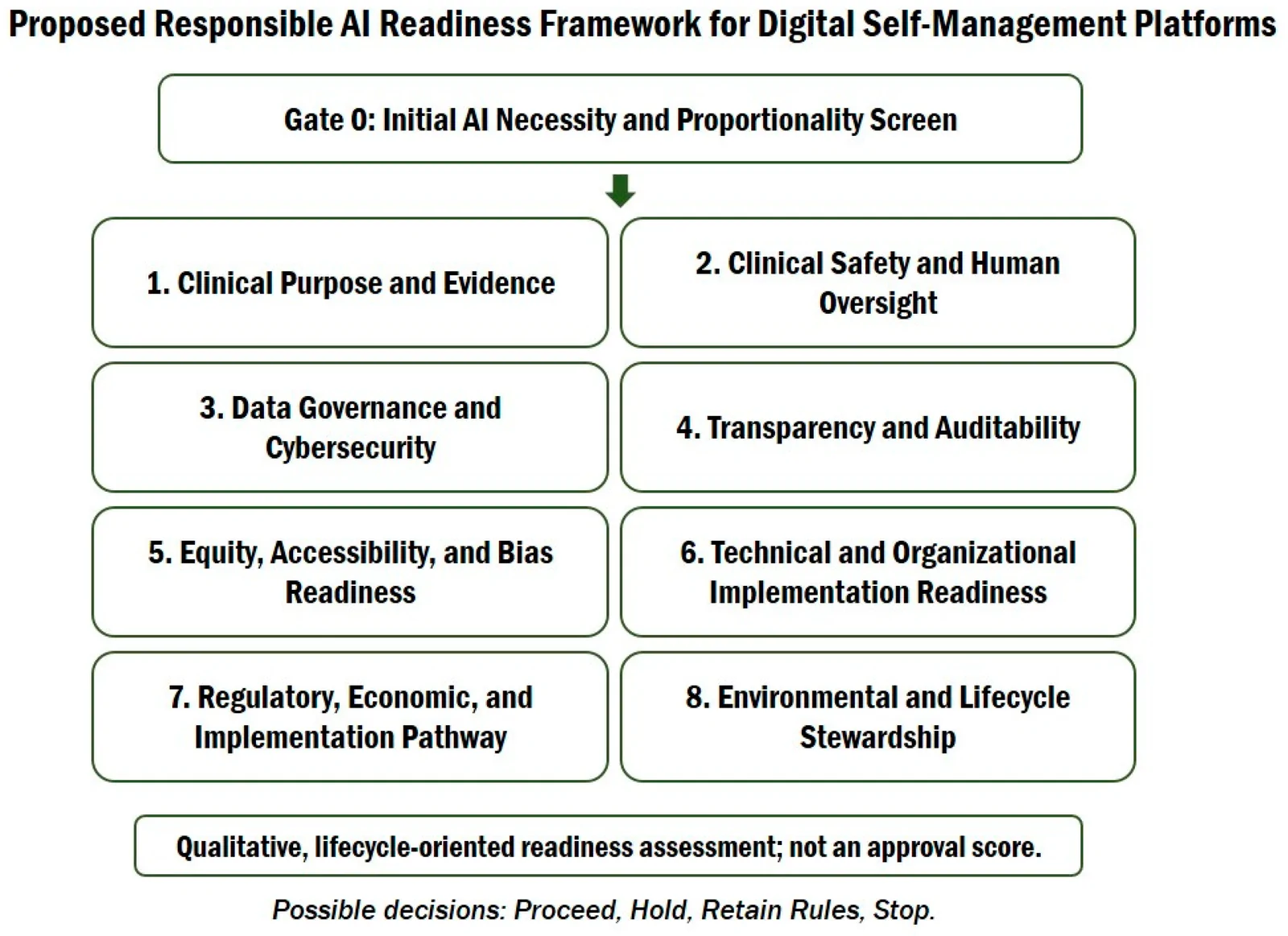
Proposed Responsible AI Readiness Framework for digital self-management platforms. Gate 0 provides an initial AI necessity and proportionality screen before assessment across eight readiness domains. Readiness is qualitative and lifecycle-oriented; it is not an approval score.

The Responsible AI Readiness Framework comprises an AI necessity and proportionality gate followed by eight readiness domains (Figure 1). Table 3 summarizes the core readiness question and the minimum evidence expected in each domain.

### 4.1. Gate 0: Initial AI Necessity and Proportionality Screen

Before model choice, teams should define the decision AI would support, the failure it would avoid, and the prespecified incremental benefit over explicit rules. If rules are clinically acceptable and traceable, the burden of proof lies with the proposed AI component.

Gate 0 also requires representative data, reliable labels, governance, monitoring, and proportionate resources. Possible decisions are proceed, hold for evidence, retain the rule-based baseline, or stop the proposed AI use.

### 4.2. Domain 1: Clinical Purpose and Evidence

The platform needs a bounded intended purpose, target population, use environment, claims hierarchy, and evidence linking content to a plausible rationale. Self-management software should not drift into diagnosis or treatment through wording, scores, or automated advice.

### 4.3. Domain 2: Clinical Safety and Human Oversight

Safety requires explicit red flags, contraindications, medication boundaries, escalation and adverse-event procedures, limits of automated advice, and named responsibility for reviewing, overriding, suspending, or investigating system behavior.

### 4.4. Domain 3: Data Governance and Cybersecurity

Data governance covers lawful purpose, minimization, consent, provenance, quality, access, retention, deletion, portability, incident response, and accountability. Cybersecurity must cover the product lifecycle, including access control, encryption, dependencies, backup, logging, and incident response [12].

### 4.5. Domain 4: Transparency and Auditability

Stakeholders should be able to determine what the system does, why an output was generated, which version was active, and what changed. Explicit rules are a useful traceable baseline, although traceability alone does not establish validity.

### 4.6. Domain 5: Equity, Accessibility, and Bias Readiness

Mobile platforms can reduce some access barriers while excluding users with limited digital access, disability, low literacy, language differences, older devices, or limited data. Accessibility requires evaluation with representative users, not inference from smartphone availability.

### 4.7. Domain 6: Technical and Organizational Implementation Readiness

Technical readiness includes maintainable architecture, modularity, version control, testing, interoperability, documentation, and dependency management. Organizational readiness includes accountable ownership, staffing, governance, support, procurement, workflow integration, and monitoring.

### 4.8. Domain 7: Regulatory, Economic, and Implementation Pathway

A viable pathway aligns intended use, evidence strategy, regulatory classification, reimbursement context, workflow, maintenance cost, and service ownership. Scalability does not remove the costs of evaluation, cybersecurity, support, updates, training, or post-market monitoring.

### 4.9. Domain 8: Environmental and Lifecycle Stewardship

Digital health may reduce some travel or material use while increasing device, network, storage, computation, and replacement demands. Environmental effects require an explicit system boundary and should not be inferred from remote delivery [9,10].

For the proposed AI, compare the same task and workload against the rule baseline, document the relevant system boundary and assumptions, measure energy or carbon where feasible, and avoid generic per-query estimates as product evidence. No environmental benefit should be claimed without measurement [8,9,10].

### 4.10. Operational Use and Decision Rules

The unit of assessment is a versioned digital platform together with one prespecified proposed AI function, a bounded intended use, target population, use setting, and regulatory context. Evidence within each domain is classified as met, partially met, unmet, or not applicable. “Met” indicates that the required evidence or governance activity is documented and attributable to a responsible owner. “Partially met” indicates that relevant evidence exists but remains incomplete or has not been independently verified. “Unmet” indicates that the required evidence or governance activity is absent. “Not applicable” requires an explicit justification based on the intended use and risk.

Admissible evidence may include versioned software specifications, content–evidence maps, safety and data-governance documents, technical test reports, user-evaluation records, regulatory documents, and prospective study evidence. Unsupported verbal assurances alone are not considered sufficient evidence.

Application should involve assessors with expertise proportionate to the intended function and risk, including relevant clinical, technical, data-governance, implementation, and regulatory expertise. Conflicts of interest should be disclosed, and independent assessment is preferable when conclusions are used beyond internal development.

Decisions are qualitative rather than score-based. Proceed means that no blocking condition prevents progression to the next evidence-generation stage; hold means that potentially remediable evidence is missing; retain rules means that no clinically or operationally meaningful incremental benefit of AI has been prespecified over the non-AI baseline; and stop means that the proposed AI function presents an unacceptable or unmanageable safety, legal, equity, organizational, or resource burden. No aggregate readiness score is calculated because strength in one domain cannot compensate for an unmet blocking condition in another. These qualitative decision rules were applied to the Sokcare worked example described below.

## 5. Author-Conducted Worked Example Using Sokcare

### 5.1. Current Platform and Workflow

**Figure 2:**
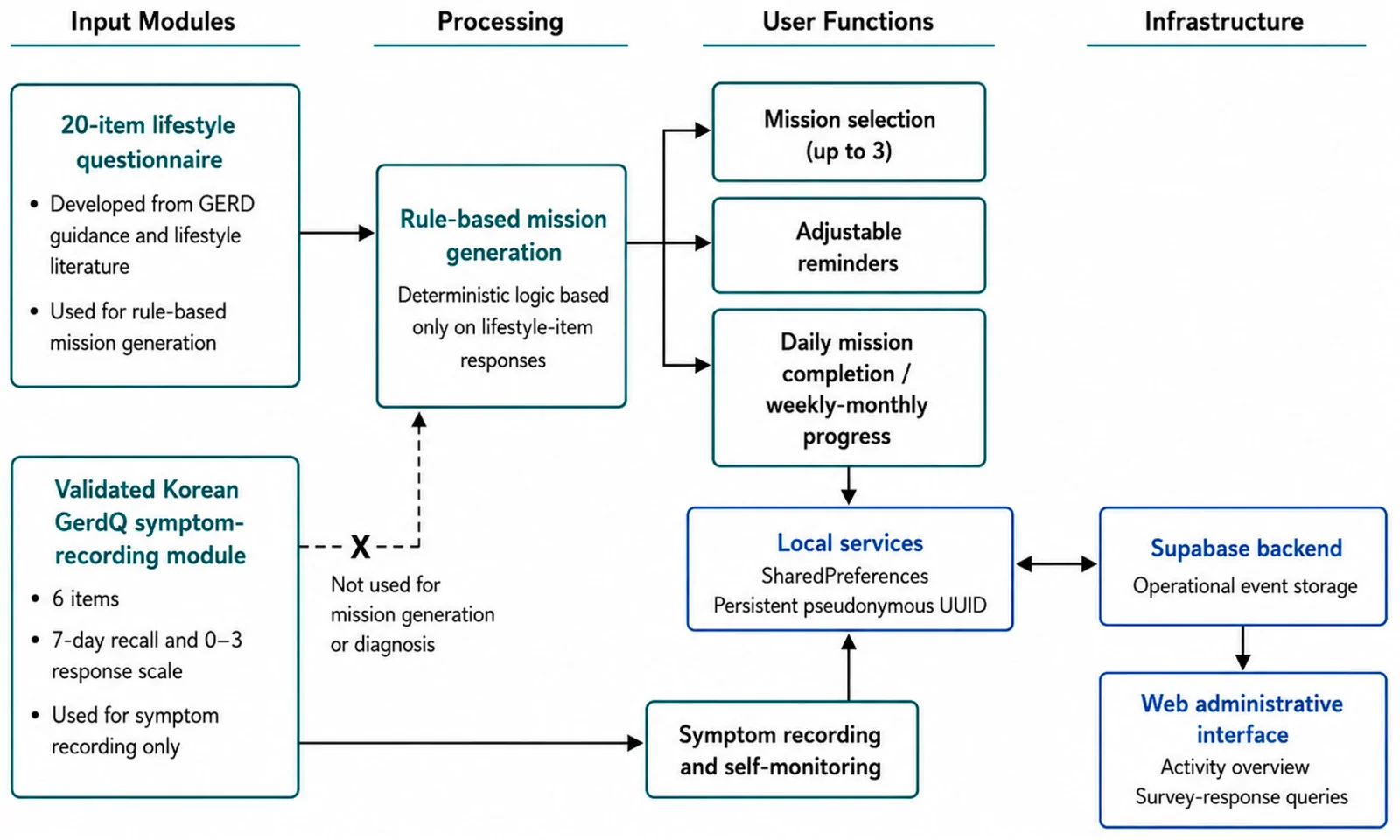
Current Sokcare architecture and data flow. The case includes explicit rules and operational components but no AI model or clinical-efficacy claim.

Sokcare is a rule-based Flutter mobile platform for lifestyle self-management among adults with recurring reflux symptoms (fixed snapshot: version 1.0.5+14). As shown in Figure 2 and Table 4, onboarding leads to a 20-item lifestyle questionnaire and a separate six-item symptom-recording module; deterministic rules generate mission candidates, users may select up to three, and adjustable reminders and progress views support completion. The prototype used the published Korean GerdQ structure, but electronic-use licensing had not been completed; the module is therefore not intended for further public or research use until authorization is confirmed. The six-item module retained the Korean GerdQ’s 7-day recall period, 0–3 response coding, reverse scoring of negatively predictive items, and internal total-score calculation; no total or diagnostic threshold was displayed to users [50,51].

The GerdQ module was used only for symptom recording and self-monitoring. Its responses and total score did not diagnose or classify users, determine treatment, provide medication advice, trigger escalation, or generate or prioritize missions [50,51].

The 20 lifestyle items are linked to mission rationale, text, and images. Mission candidates are generated only from questionnaire responses through explicit deterministic rules; Supplementary Methods S2, Algorithm S1, and Decision Table S1 provide the full reproducible specification.

Users can change default and mission-specific reminders. The local state is held in SharedPreferences and a pseudonymous UUID supports operational event storage in Supabase; no service-user data, symptom scores, dashboard metrics, or participant outcomes were analyzed here.

**Figure 3:**
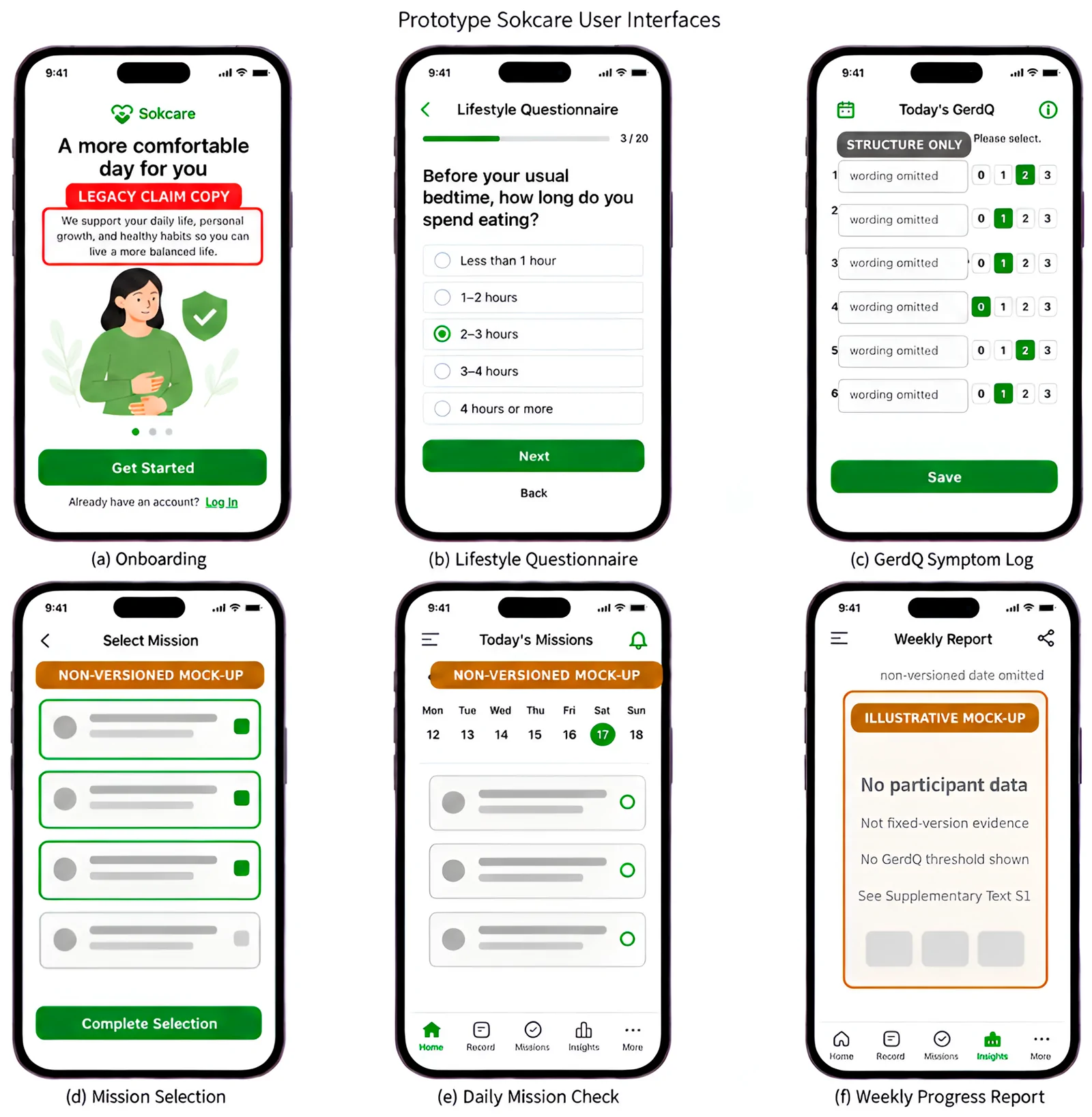
Interface design record with content-status annotations: (**a**) onboarding with legacy symptom-improvement and personalization wording; (**b**) lifestyle questionnaire; (**c**) GerdQ symptom-log structure, with item wording removed; and (**d**–**f**) non-versioned mock-ups for mission selection, daily checking, and weekly reporting. (**d**–**f**) show layout only and must not be used to infer version 1.0.5+14 content or functionality; Supplementary Table S1 is the authoritative fixed mission record. The GerdQ module did not influence mission generation or clinical classification, and the fixed version did not display a GerdQ total or diagnostic threshold. Values in the source mock-ups were illustrative, not participant outcomes. English transcription and panel-level boundaries are provided in Supplementary Text S1.

The prototype is private while content and safety functions are reviewed. It has no dedicated red-flag detection/escalation pathway or real-time monitoring, and it does not diagnose GERD, modify medication, or issue urgent recommendations. Before any public release, it needs a clinically reviewed user-facing safety notice and referral instructions that explicitly cover chest pain, dysphagia, gastrointestinal bleeding, persistent vomiting, unexplained weight loss, and suspected anemia, as well as stating that the service does not provide emergency or real-time clinical monitoring. Figure 3f is a non-versioned mock-up: the fixed version displayed neither a GerdQ total nor a diagnostic threshold.

### 5.2. Clinical Content, Safety Boundary, and Formative Consultation

The 20 lifestyle items cover behavior and context relevant to reflux self-management, but evidence strength varies. Item-level review classified 18 fixed mission statements as Revise and 2 as Retain; this is a content-audit count, not an effect estimate. Supplementary Table S1 preserves the fixed wording, evidence boundary, and proposed future specification [19,20,21,22,23,52,53,54,55,56]. These future specifications were not deployed in version 1.0.5+14 and have not undergone formal gastroenterology or behavioral-science content validation.

Eight formative consultation roles highlighted bounded claims, evidence provenance, user burden, privacy and cybersecurity, separation of current and future functions, and staged evaluation. These records informed the case description but are not an independent validation or endorsement. BCTTv1 was used only as descriptive vocabulary, not as a formal coding or efficacy study [43].

Legacy onboarding language promising symptom improvement through “personalized missions” creates a claim-governance risk because it implies benefit and personalization without supporting outcome evidence. It should be replaced with a bounded self-management description; as Figure 3 is not version-linked, this documents a design-level risk rather than the proving display shown in version 1.0.5+14.

### 5.3. Worked-Example Findings

The worked example generated provisional domain-level classifications using the qualitative rules in Section 4.10 rather than an aggregate score. Available artifacts suggested foundations in a bounded self-management purpose, explicit item-to-mission links, user choice, adjustable reminders, modular architecture, and pseudonymous operational records. These assets make the rule behavior inspectable. The resulting judgments remain author-generated and have not been independently validated.

The platform is not ready for AI-enabled clinical claims or healthcare implementation. Clinical and safety gaps include the absence of prospective validation and a dedicated red-flag symptom detection and escalation pathway. Technical and governance gaps include representative model-development data, accessibility evaluation, independent cybersecurity testing, decision-log governance, and bias and drift plans. Implementation gaps include workflow integration, cost-effectiveness evidence, reimbursement planning, and environmental measurement. The provisional decision from this worked example was therefore to retain the rule-based baseline and defer AI development while the identified foundational gaps are addressed. These provisional findings are summarized in Table 5.

## 6. Staged Transition Pathway

**Figure 4:**
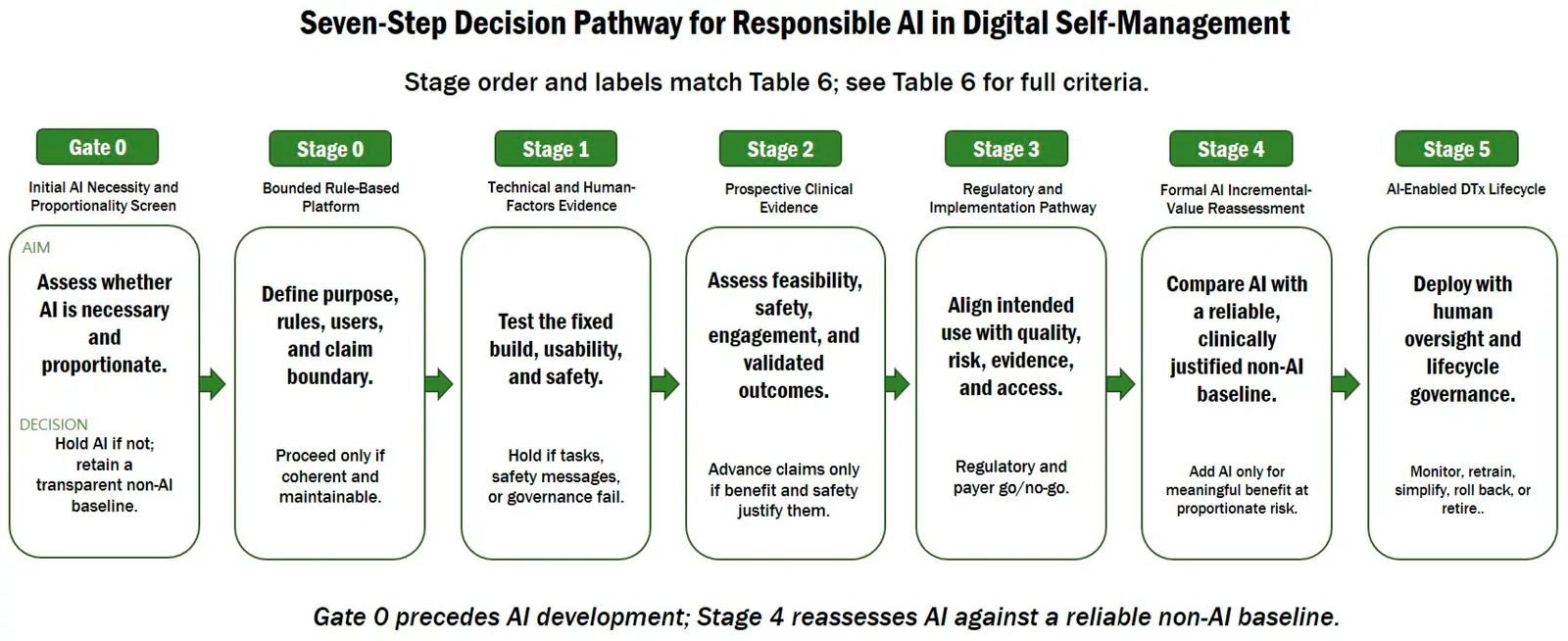
Seven-step staged pathway from an initial AI necessity screen to an AI-enabled DTx lifecycle. Stage labels, sequence, primary objectives, and decision statements match Table 6 exactly. Gate 0 is the initial pre-model screen; Stage 4 is the later comparator-based reassessment. Progression is conditional, and retaining the non-AI baseline is an explicit responsible decision.

The staged pathway makes progression conditional: a platform may remain a bounded rule-based service, proceed to an evaluated digital intervention, or develop toward regulated DTx. AI is considered only after underlying clinical, technical, governance, equity, implementation, economic, and environmental foundations are demonstrated (Figure 4; Table 6).

Gate 0 is iterative. After a stable non-AI baseline has been evaluated proportionately to its intended claim, a later reassessment asks whether AI has demonstrated incremental value.

Interpretability alone does not make a rule baseline adequate; before formal AI comparison, it must be technically reliable, clinically justified, and prospectively evaluated with relevant benefit and harm outcomes.

## 7. Discussion

### 7.1. Principal Conceptual Contribution

This paper reframes responsible AI readiness as a pre-adoption clinical-governance and healthcare-implementation problem. Existing AI guidance provides essential principles for models and AI-enabled devices [3,4,5,6,7], but early digital-health teams also need a structured way to decide whether AI should be introduced. The proposed framework adds an explicit necessity and proportionality gate, integrates eight readiness domains, and treats responsible non-adoption as a legitimate outcome.

The framework also separates AI readiness from AI performance. A platform is not ready merely because it stores structured data or can call an external model. Readiness requires a clinically meaningful use case, valid evidence, safety routing, human accountability, lawful and representative data, accessibility, maintainable operations, a feasible health-system implementation model, a viable regulatory and economic pathway, and lifecycle environmental stewardship. This broader definition connects technical innovation with safe healthcare delivery, implementation science, regulatory planning, and accountable lifecycle governance.

The present framework therefore does not claim novelty for each domain in isolation. Its narrower contribution is procedural: within the reviewed set, no single framework was designed specifically to decide whether an early rule-based self-management platform should add AI by using a fixed non-AI comparator, prespecified incremental-benefit criteria, and bounded proceed, hold, retain-rules, or stop outputs. The framework links that decision to clinical claims and safety escalation, data and oversight prerequisites, implementation ownership, regulatory–economic progression, equity, and environmental proportionality. It is intended to complement rather than replace the source frameworks.

### 7.2. Implications of the Sokcare Case

The Sokcare worked example illustrates the potential value of a transparent rule-based baseline. Its explicit rules make the item–mission relationship visible and allow the user to choose a limited set of actions. This is an asset for debugging, content governance, and future comparative evaluation. It also shows why an AI label would currently add more claim burden than evidence value. No defined use case has yet shown that machine learning would outperform the current rules in a clinically meaningful way.

The worked example also illustrates why transparency alone is insufficient. A rule can be understandable yet clinically unvalidated; a pseudonymous backend can exist without independent security evidence; a mobile interface can be flexible without being accessible to underserved users; and a technically scalable platform can lack safety escalation, organizational ownership, workflow integration, reimbursement, or lifecycle evidence. The readiness framework prevents these partial strengths from being mistaken for global readiness.

Most concretely, the content audit classified 18 of 20 fixed mission statements as requiring revision and only 2 as retainable. This count is not clinical-outcome evidence, but it provides a quantitative check against assuming that inspectable rules are clinically appropriate. The legacy wording in the design record exposed a parallel design-level claim-boundary risk with potential regulatory implications. Together, these findings show how a readiness review can detect defects in both deterministic content and user-facing claims before AI is considered.

### 7.3. Implications for Developers and Healthcare Innovators

For developers, the framework supports claim-matched, staged development: bounded self-management functions may refine workflows, whereas therapeutic claims require a fixed intervention, prospective evidence, risk management, implementation ownership, and a maintainable service model. AI should be deferred when its incremental value, data, monitoring, or proportionality case is inadequate.

### 7.4. Implications for Health Systems, Regulators, and Funders

Health systems, regulators, and funders can use the domains as a shared staged-evidence language, distinguishing a prototype from an evaluated intervention, regulated DTx, or AI-enabled medical product. Funding and procurement should require claim-matched safety, ownership, accessibility, evidence, and lifecycle milestones rather than AI inclusion alone.

### 7.5. Healthcare Delivery, Equity, and Lifecycle Implications

Potential healthcare contribution remains a testable chain: evidence-bounded self-management may improve appropriate daily behavior and then symptoms or well-being. The present case establishes neither health effect nor SDG 3 contribution; it identifies what must be evaluated, including access barriers and differential outcomes [2].

### 7.6. Limitations and Research Agenda

This targeted synthesis is neither exhaustive nor a formal consensus process, and the eight domains have not been validated as a measurement instrument. Sokcare is a single author-associated Korean rule-based case; secondary coauthor review improves internal consistency but is not independent validation. Future studies should use prespecified evidence sources and multidisciplinary Delphi refinement, assess multiple independent rule-based and AI-enabled platforms with at least two independent raters, and report inter-rater agreement and reasons for disagreement.

The case included no participant outcomes, independent usability or cybersecurity testing, health-system implementation testing, or economic and environmental measurement. Formative consultation was not a standardized expert-rating study; proprietary code and operational security were not independently audited. Regulatory, reimbursement, and care-integration pathways remain jurisdiction-specific, and independent assessors without financial, developmental, or intellectual-property relationships are needed.

Future research should refine the domains through multidisciplinary consensus, test reliability, compare product types, and examine whether readiness predicts safer evaluation, implementation, sustained use, equity, cost, or environmental burden. For Sokcare, the priorities are safety and referral guidance, technical and accessibility testing, prospective usability and feasibility research, validated outcomes, implementation and economic evaluation, and only then a prespecified AI-versus-rule test.

## 8. Conclusions

Digital self-management platforms should not treat AI adoption as automatic progress. This framework begins with AI necessity and proportionality, then assesses eight domains spanning clinical evidence and safety, data and transparency, equity, implementation, regulatory–economic pathways, and lifecycle stewardship.

When applied to Sokcare, the framework identifies a transparent rule baseline but insufficient foundations for AI-enabled clinical claims or healthcare integration. The appropriate strategy is to retain the baseline, close safety and evidence gaps, and evaluate staged progression. This single author-conducted example does not establish cross-platform validity; responsible readiness includes deferring or rejecting AI when a simpler system is safer, more governable, and more proportionate.

## Figures and Tables

**Table 1:** Conceptual foundations used to construct the Responsible AI Readiness Framework.

Source Family	Core Contribution	Readiness-Framework Use
WHO digital health and health-system strategy [1]	Equitable access; health-system strengthening; governance; integration of financial, organizational, human, and technical resources	Health-system fit, equity, and organizational readiness
mHealth implementation, sustainability, and human-centered design [38,39]	User fit; adoption; maintenance; health-system and lifecycle consequences	Technical, organizational, economic, social, and environmental readiness
WHO AI ethics and regulatory documents [3,4]	Autonomy; safety; transparency; accountability; inclusiveness; regulatory lifecycle	Safety, human oversight, transparency, equity, lifecycle governance
NIST AI RMF and FUTURE-AI [5,6]	Govern–Map–Measure–Manage; fairness; universality; traceability; usability; robustness; explainability	Cross-cutting risk functions and auditability
IMDRF AI/ML, SaMD, and cybersecurity documents [7,11,12]	Representative data; software engineering; human–AI teams; clinical evaluation; cybersecurity lifecycle	Evidence, technical quality, oversight, cybersecurity, monitoring
Green and environmentally responsible AI [8,9,10]	Compute efficiency; resource conservation; environmental lifecycle effects	AI proportionality and environmental stewardship
DTx evidence and Korean regulatory context [13,14,15]	Intended use; clinical evidence; lifecycle RWE; market and reimbursement pathway	Clinical purpose, regulatory progression, economic viability
Digital-intervention and implementation frameworks [36,37,40,41,42,43,44,45,46,47,48,49]	Human-centered design; intervention specification; implementation complexity; multidimensional assessment; staged reporting	Case application, health-system integration, and staged evaluation

**Table 2:** Comparative positioning of the proposed framework relative to established digital-health and AI frameworks.

Framework or Guidance	Primary Stated Scope	Contribution Used in the Present Framework	Pre-Adoption Question Not Fully Operationalized
WHO digital health and AI guidance [1,3,4]	Health-system strategy together with ethical and regulatory principles for AI in health.	Governance, autonomy, safety, accountability, inclusion, equity, and lifecycle orientation.	A platform-level test of whether AI adds value over a fixed non-AI baseline before model development is not operationalized.
NIST AI RMF [5]	Cross-sector AI risk management across design, development, use, and evaluation.	Govern–Map–Measure–Manage functions; risk ownership, documentation, measurement, and monitoring.	Clinical claim boundaries, clinical safety-escalation ownership, and task-specific incremental benefit over deterministic rules require health-domain specification.
FUTURE-AI [6]	Development and deployment of trustworthy AI tools in healthcare.	Fairness, universality, traceability, usability, robustness, and explainability across the AI lifecycle.	It guides trustworthy AI development and deployment rather than primarily deciding whether a functioning non-AI platform should add AI.
NASSS [48]	Adoption, nonadoption, abandonment, scale-up, spread, and sustainability of health technologies.	Legitimacy of nonadoption; contextual complexity; organizational and system fit.	Nonadoption is explicit, but an AI-specific necessity test with a fixed rule comparator, data and label prerequisites, and prespecified incremental benefit is not provided.
MAST [49]	Preceding considerations, multidimensional assessment, and transferability of telemedicine applications.	Clinical, patient, economic, organizational, socioethical, and transferability perspectives.	AI-specific data and model governance and a pre-development rule-versus-AI necessity decision lie outside its telemedicine-assessment purpose.
CeHRes Roadmap 2.0 [40,41]	Human-centered development, implementation, and evaluation of eHealth technologies.	Context, stakeholder involvement, iterative evaluation, and implementation planning.	It does not define an AI-specific go, hold, retain-rules, or stop gate or a task-matched rule-versus-AI resource comparison.
DTx RWE Framework [14]	Evidence-based DTx design, development, testing, deployment, and monitoring.	Iterative evidence generation and lifecycle monitoring.	It does not specifically determine whether AI should be added to a functioning rule-based platform before model development.

**Table 3:** Eight readiness domains, core questions, and minimum evidence before AI adoption.

Domain	Core Readiness Questions	Examples of Minimum Evidence
1. Clinical purpose and evidence	Is the purpose bounded? What decision would AI change? Are valid outcomes and labels available?	Intended-use statement; evidence map; prespecified outcomes; claim–evidence matrix
2. Clinical safety and human oversight	Which harms require escalation? Who reviews, overrides, pauses, and investigates?	Safety requirements; red-flag symptom and referral requirements; oversight roles; incident and adverse-event plan
3. Data governance and cybersecurity	Are data lawful, minimal, representative, secure, and traceable across the lifecycle?	Data inventory; provenance; access and retention policies; security testing; breach response
4. Transparency and auditability	Can outputs, versions, changes, and limitations be inspected and explained?	Rule/model cards; decision logs; version history; uncertainty and limitation statements
5. Equity, accessibility, and bias readiness	Who may be excluded or harmed? Are intended populations represented and accessible?	Accessibility testing; subgroup plan; representativeness and differential-performance analysis
6. Technical and organizational implementation readiness	Can the system be maintained, monitored, integrated into care pathways, supported, and retired?	Architecture and test plan; staffing; clinical and operational workflows; update, rollback, and retirement process
7. Regulatory, economic, and implementation pathway	Are intended use, regulatory evidence, reimbursement, workflow integration, and lifecycle costs viable?	Regulatory strategy; health-economic plan; implementation and service model; lifecycle budget
8. Environmental and lifecycle stewardship	Is computational complexity proportionate, and are environmental effects measured?	Task-matched rule-versus-AI benchmark; defined system boundary; inference, storage, and data-transfer workload; measured energy or transparent proxy; carbon-intensity assumptions; hardware and hosting inventory; retirement criteria

**Table 4:** Current Sokcare components and their bounded interpretation in the case application.

Component	Current Implementation	Interpretation Boundary
Lifestyle module	Twenty literature-informed lifestyle items with 1–5 response levels and linked mission identifiers	Developed for rule-based mission generation; not a validated diagnostic or risk scale.
Symptom-recording module	Six-item symptom-recording module implementing the structure and scoring logic of the validated Korean GerdQ	Electronic-use authorization remains to be confirmed. The module was for symptom recording only and was not used for diagnosis, clinical classification, treatment decisions, medication advice, escalation, or mission generation.
Mission library	Twenty lifestyle-item-linked rationales, missions, and images	Behavior-support content only. The 18 missions classified as Revise in Supplementary Table S1 should not be used in further public or clinical evaluation until a version-controlled content review, including gastroenterology and behavioral-science review, is complete.
Candidate rule	Explicit deterministic sequence based exclusively on responses to the 20 lifestyle items, with deduplication	Transparent mission prioritization; GerdQ responses and scores do not influence mission generation or selection; no clinical prediction.
User agency	Selection of up to three missions	Reduces automated prescription; optimal mission number and intervention dose have not been established.
Reminders	Two general defaults plus user-adjustable mission reminders	Engagement mechanism; effect on adherence or clinical outcomes has not been evaluated.
Self-monitoring and reports	Daily mission-completion records, weekly/monthly completion summaries, and separate GerdQ response records; the report in Figure 3f is a non-versioned design mock-up	Supports behavior and symptom tracking only. The fixed version did not display a GerdQ total or diagnostic threshold, and mock-up values are not outcome evidence.
Backend and administration	Pseudonymous UUID and operational event tables	No participant-level user data were analyzed; security has not been independently audited
Red-flag symptom detection and escalation	No dedicated GERD red-flag detection or escalation pathway was present in the fixed version	A clinically reviewed user-facing safety notice and referral instructions covering chest pain, dysphagia, gastrointestinal bleeding, persistent vomiting, unexplained weight loss, and suspected anemia are minimum requirements before further public use. Automated detection, monitored escalation, and a named clinical recipient would require additional governance and should be determined by the intended function and risk of a future regulated intervention.
AI	No machine-learning or generative model	Transparent rule-based baseline for readiness assessment.

**Table 5:** Author-conducted worked example of the proposed framework applied to Sokcare.

Domain	Provisional Status and Evidence Basis	Main Readiness Gap	Provisional Decision and Next Step
Gate 0: Initial AI necessity and proportionality screen	Initial screen completed. Explicit, reviewable rules currently perform lifestyle-based mission generation (A1–A2).	No prespecified AI use case, representative training dataset, or clinically or operationally meaningful incremental-benefit criterion.	Retain rules and hold AI development. Define a testable AI use case, comparator, and added-value criterion before model development.
1. Clinical purpose and evidence	Partially met. The intended use is bounded to lifestyle self-management, and the content and symptom-recording functions are documented (A1–A3). The item-level audit classified 18 of 20 fixed missions as Revise and 2 as Retain.	Most fixed mission statements exceeded their evidence boundaries. Legacy onboarding copy in the non-version-linked design record implied symptom improvement and personalization without outcome evidence. Electronic-use authorization for the Korean GerdQ remains to be confirmed, and no prospective clinical-effect data were analyzed.	Hold diagnostic and therapeutic claims. Revise the 18 missions, withdraw or replace the legacy claim wording, confirm GerdQ authorization, complete content review, and conduct usability and prospective evaluation.
2. Clinical safety and human oversight	Unmet. User choice and a non-diagnostic claim boundary are present, but these do not constitute a clinical safety pathway (A1, A3).	No dedicated GERD red-flag symptom detection and escalation pathway, real-time clinical monitoring, or adverse-event process was present in the fixed version.	Hold further public or clinical use until minimum safety requirements are addressed. Add a clinically reviewed safety notice, referral instructions, and a clear statement that the service is not monitored in real-time.
3. Data governance and cybersecurity	Partially met. Pseudonymous UUID use, local storage, and backend data flow are documented (A1, A4).	No independent row-level security, access, vulnerability, penetration, retention/deletion, or lifecycle incident-response assessment was reported.	Hold expansion or secondary use of data. Complete a data inventory, access audit, security testing, retention/deletion policy, and incident-response documentation.
4. Transparency and auditability	Partially met. The deterministic rules and item–mission links are inspectable (A1–A2).	Formal rule and content approval, version history, change control, and decision-log governance are incomplete.	Proceed with documentation only. Version the rules, content, rationale, and changes, and preserve the fixed rule baseline for future comparison.
5. Equity, accessibility, and bias readiness	Unmet because it was not evaluated. Prototype screens and adjustable reminders may support flexibility, but no representative accessibility evaluation was performed (A3).	No testing involving older adults, people with disabilities, low literacy, limited connectivity, language needs, or underserved groups.	Make no equity or accessibility claims at this stage. Conduct an accessibility audit and participatory evaluation with relevant intended users.
6. Technical and organizational implementation readiness	Partially met. Cross-platform architecture and backend and administrative functions are available (A1, A4).	No formal maintenance owner, interoperability plan, clinical or service workflow, staffing model, user support, rollback process, or retirement plan.	Hold healthcare integration. Establish accountable ownership, operational workflows, testing, support, dependency management, rollback, and retirement processes.
7. Regulatory, economic, and implementation pathway	Unmet for therapeutic or regulated progression. The low-complexity prototype provides an intended-use starting point, but not a DTx evidence package (A1–A3).	Legacy symptom-improvement and personalization wording may imply a therapeutic or individualized intervention claim. No formal regulatory classification, quality-management system, clinical evaluation package, cost-effectiveness evidence, reimbursement pathway, procurement plan, or care-integration model was established.	Retain bounded self-management positioning and hold DTx claims. Remove or replace the legacy wording, define the intended use and regulatory classification, and then specify lifecycle cost, QMS, evidence, market-access, and reimbursement requirements.
8. Environmental and lifecycle stewardship	Unmet because it was not measured. Rule execution is technically simple, but no product-specific resource or environmental assessment was performed (A1, A4).	No energy, carbon, hosting, device-lifecycle, data-transfer, storage, or counterfactual-care measurements.	Make no environmental-benefit claim. Conduct a task-matched benchmark of the rule baseline and any proposed AI component before AI adoption.

All classifications were generated by the authors and should be interpreted as a worked example rather than independent certification. Artifact keys are as follows: A1, fixed Flutter source snapshot version 1.0.5+14; A2, item–mission content map and deterministic rule specification; A3, prototype interface screens; and A4, backend and data-flow documentation. No aggregate readiness score was calculated.

**Table 6:** Seven-step staged pathway. Stage labels, sequence, primary objectives, and decisions correspond exactly to Figure 4; this table additionally lists the required evidence or output.

Stage	Primary Objective	Required Evidence/Output	Decision
Gate 0: Initial A Inecessity and proportionality screen	Decide whether AI should be considered before model development.	Defined decision and failure mode; transparent non-AI comparator; plausible patient-relevant or operational added-value hypothesis; preliminary risk, burden, and resource rationale.	Hold AI if necessity or proportionality is unproven; continue with a transparent non-AI baseline.
Stage 0: Bounded rule-based platform	Clarify purpose, content, rules, users, and claim boundary.	Fixed specification; evidence map; privacy and security requirements; clinical-governance and maintenance owner.	Proceed only if the low-risk self-management function is coherent and maintainable. Low-risk administrative, reminder, or engagement functions that do not alter clinical recommendations, risk classification, or behavior-support decisions do not automatically require a DTx pathway.
Stage 1: Technical and human-factors evidence	Verify the fixed build and usability before clinical claims.	Unit, integration, and end-to-end tests; accessibility; usability; workflow and safety-message testing; cybersecurity; content review.	Hold if users cannot complete tasks, safety messages fail, or governance is incomplete.
Stage 2: Prospective clinical evidence	Evaluate feasibility, safety, engagement, and validated outcomes under ethics oversight.	Registered protocol; validated outcomes; harms; retention; subgroup and process analysis.	Proceed to a therapeutic claim only if benefit and safety justify it.
Stage 3: Regulatory and implementation pathway, when applicable	Align intended use with quality, risk, clinical evaluation, and market-access requirements.	QMS; risk file; clinical evaluation; human-factors file; implementation, reimbursement, and market-access strategy.	Regulatory and payer go/no-go decision.
Stage 4: Formal AI incremental-value reassessment	Compare the proposed AI function with a technically reliable and clinically justified non-AI baseline.	Technically reliable and clinically justified non-AI baseline; prespecified patient-relevant benefit and harm outcomes; representative data; subgroup analysis; and an appropriate comparator, including usual care or a human-supported workflow where relevant.	Add AI only if prespecified patient-relevant or operational benefits are meaningful and the added risks and burdens are proportionate.
Stage 5: AI-enabled DTx lifecycle	Deploy only with human oversight and continuous governance.	Monitoring, drift, incidents, updates, rollback, equity, cost and environmental indicators; retirement criteria.	Continue, retrain, simplify, roll back, or retire according to monitored benefit–risk.

## Data Availability

The proposed framework, case-application matrix, and comparative positioning are contained in this article. The targeted search details, item–mission evidence map, source-to-domain audit trail, and comparison basis are provided in the Supplementary Materials (Supplementary Methods S1 and S2, Algorithm S1, Decision Table S1, Supplementary Text S1, Tables S1–S6). No participant dataset was analyzed. The complete application source code and operational configurations are not publicly available because they contain proprietary and security-sensitive materials.

## Supplementary Materials

The following supporting information can be downloaded at: https://www.mdpi.com/article/10.3390/healthcare14183061/s1, Supplementary Methods S1, targeted integrative synthesis search and consolidation procedure; Supplementary Methods S2, deterministic mission-candidate generation; Algorithm S1, reproducible pseudocode for the fixed rule comparator; Decision Table S1, deterministic handling of edge cases; Supplementary Text S1, English functional transcription of the Sokcare interface panels shown in Figure 3; Table S1, Sokcare lifestyle item–mission review and evidence-bounded revision; Table S2, source identification and selection summary; Tables S3A and S3B, domain-level derivation audit trail and complete source-to-element mapping for the 56 retained sources; Table S4, basis and interpretation boundary for the comparative positioning in Table 2; Table S5, reusable qualitative assessment form; and Table S6, Sokcare case-evidence register (artifact keys A1–A4).
