# Supplementary material for: A Responsible AI Readiness Framework for Digital Self-Management Platforms: Illustrative Application to a Rule-Based GERD Platform
Source: Healthcare (Basel). 2026 Sep 17;14(18):3061. doi: 10.3390/healthcare14183061 (PMC13606674; doi:10.3390/healthcare14183061)
Supplement: Supplementary file 1 [file healthcare-14-03061-s001.zip › healthcare-4515399-supplementary.pdf]

Supplementary Methods S1. Targeted integrative synthesis search and consolidation procedure

Search sources and date. Targeted searches were completed on 4 August 2026 in PubMed/MEDLINE and on the official websites of WHO, the United Nations, NIST, IMDRF, and the Korean Ministry of Food and Drug Safety (MFDS). Backward citation checking and targeted supplementation from key guidance, framework, clinical, and case-boundary sources were used to identify additional material.

Complete PubMed/MEDLINE search strategy. The following three search blocks reproduce the strings recorded for the revision. Retrieval counts are the contemporaneous counts recorded on 4 August 2026; later reruns may differ as PubMed is updated.

Search 1 (423 records): ("digital health"[Title/Abstract] OR mHealth[Title/Abstract] OR "digital therapeutics"[Title/Abstract]) AND (implementation[Title/Abstract] OR governance[Title/Abstract] OR lifecycle[Title/Abstract] OR sustainability[Title/Abstract])

Search 2 (769 records): ("artificial intelligence"[Title/Abstract] OR "health AI"[Title/Abstract]) AND (responsible[Title/Abstract] OR trustworthy[Title/Abstract] OR fairness[Title/Abstract] OR transparency[Title/Abstract] OR cybersecurity[Title/Abstract] OR environmental[Title/Abstract] OR lifecycle[Title/Abstract])

Search 3 (56 records): (GERD[Title/Abstract] OR "gastroesophageal reflux"[Title/Abstract]) AND (mobile[Title/Abstract] OR app[Title/Abstract] OR digital[Title/Abstract] OR "self-management"[Title/Abstract] OR lifestyle[Title/Abstract]). **Combined result:** 1,248 records; 51 duplicates removed; 1,197 unique PubMed records.

Selection procedure and counts. S.-Y.K. reviewed titles and abstracts and, when needed, full texts or complete official documents for direct relevance to a Gate 0 decision, one or more readiness domains, comparison-framework scope, GERD clinical boundary, or Sokcare case interpretation. Seven of the 1,197 unique PubMed records were retained directly in the final reference set; 1,190 were not retained. Nine authoritative documents identified on official websites were retained, and 40 additional sources were retained through backward citation checking and targeted supplementation, giving 56 retained sources in total. Because this was an iterative targeted synthesis rather than a systematic review, stage-specific exclusion reasons and the number of citations encountered but not retained during backward checking were not prospectively coded and are not reconstructed retrospectively.

Eligibility principles. Eligible materials were official regulatory and organizational documents, consensus statements, clinical practice guidelines, framework papers, systematic or scoping reviews, and empirical digital-health or GERD studies that informed a pre-AI decision, minimum evidence requirement, clinical-safety or implementation activity, governance requirement, or case boundary. Duplicate or superseded guidance, model-performance studies without lifecycle relevance, and disease studies that did not inform implemented content or its interpretation boundary were not retained.

Concept extraction and comparative positioning. Each retained source was abstracted into six fields: (1) decision question, (2) failure mode, (3) minimum evidence or governance artifact, (4) responsible actor, (5) lifecycle stage, and (6)

implementation or lifecycle dimension. For each framework or guidance family compared in the main text, the authors recorded its stated scope, the contribution used in the proposed framework, and the pre-adoption question that remained insufficiently operationalized. The comparison was descriptive and did not assign quality or completeness scores.

Consolidation rules. Concepts were merged only when they addressed the same blocking decision and required the same evidence owner. A domain was retained separately when omission could independently prevent progression, the domain required a distinct evidence package or accountable role, and its function was not fully subsumed by another domain. Gate 0 was kept outside the eight domains because it can retain the rule baseline, hold, or stop a proposed AI function before domain-level readiness work begins. The source-to-element mapping for all 56 retained sources is reported in Supplementary Table S3B.

Interpretation boundary. The search was targeted and iterative. It supported conceptual construction and a worked case illustration, not exhaustive evidence retrieval, effect estimation, or ranking of existing frameworks. The counts above improve traceability but should not be interpreted as a PRISMA-compliant systematic-review flow.

Reviewer roles and adjudication. S.-Y.K. performed the initial searches, concept extraction, framework comparison, and domain consolidation. During revision, J.L. independently reviewed Gate 0, the eight domain assignments, and their primary framework-source mappings. J.-A.G. independently reviewed the GERD-related clinical evidence, safety boundaries, and all 20 item-mission mappings. Disagreements were discussed and resolved by consensus; when uncertainty remained, the more conservative interpretation was retained. This internal review does not constitute external validation or an inter-rater reliability study.

Supplementary Methods S2. Deterministic mission-candidate generation

Inputs and coding. The fixed comparator used responses to 20 lifestyle items only. GerdQ items, individual GerdQ responses, and total scores were excluded from mission generation. Each lifestyle item used an item-specific ordered response code from 1 to 5 and a fixed lookup from item identifier and response code to a mission identifier. A higher implementation code was processed before a lower code; the code is an ordinal software priority and is not a universal clinical severity score. A completed questionnaire required all 20 responses.

Candidate order, ties, and duplicates. For a new candidate set, response levels were processed from highest to lowest. Within a tied response level, items were processed in ascending item-identifier order (1-20). A mapped mission was appended only if its mission identifier had not already appeared, thereby preserving first appearance. Processing stopped when three unique candidates had been collected or when all response levels had been exhausted. Fewer than three candidates could therefore be returned.

Incomplete, prior-selection, and conflict states. If any lifestyle response was missing, the submission remained incomplete; missing values did not generate candidates. When an already-open daily state was restored, the current day's selected missions were retained in their saved order, up to the maximum of three. They were not permanently carried into a later day's newly generated set. The fixed

prototype had no clinical contraindication or semantic-conflict resolver: distinct mission identifiers could coexist as candidates, and user selection was the only final limiter. This absence is a documented safety and content-governance gap rather than an implicit decision rule.

Algorithm S1. Reproducible pseudocode for the fixed rule comparator

Pseudocode

```
INPUT: response[1..20], map[item, level] -> mission_id, MAX = 3
IF any response is missing: return INCOMPLETE_SUBMISSION
IF restoring an open daily state: return saved_current_day_missions[0:MAX]
candidates = []
FOR level IN distinct response levels, sorted descending:
    FOR item IN 1..20, sorted ascending:
        IF response[item] == level:
            mission = map[item, level]
            IF mission exists AND mission not in candidates:
                append mission to candidates
                IF length(candidates) == MAX: return candidates
RETURN candidates
```

Decision Table S1. Deterministic handling of edge cases

Condition	Action	Order/tie rule	Output
Completed 20-item submission	Generate candidates from response codes only.	Descending response code; ascending item ID within a tie.	0-3 unique mission IDs
Missing response	Block completed submission; do not map the missing item.	No imputation and no GerdQ substitution.	INCOMPLETE_SUBMISSION
Duplicate mission ID	Keep the first occurrence and discard later duplicates.	First appearance is defined by level, then item ID.	Stable de-duplicated order
Fewer than three candidates	Continue with the next-lower response code until exhausted.	Do not invent or randomly fill candidates.	Return fewer than three if necessary
Restored current-day state	Load saved current-day missions in their saved order.	Maximum three; no forced next-day carry-over.	Saved daily selection
Distinct but potentially competing missions	Retain both as candidates if reached before the limit.	No automated contraindication or clinical conflict resolution.	User selection; clinical review required before future use
GerdQ response or score	Ignore for mission generation and prioritization.	No diagnostic threshold or score-based branch.	No effect on output

Supplementary Text S1. English functional transcription of the Sokcare interface panels shown in Figure 3

Panel A. Onboarding. App name: “Sokcare.” Tagline: “A more comfortable day, together with Sokcare.” Explanatory text: “Sokcare helps improve gastroesophageal reflux symptoms through symptom recording, personalized missions, and lifestyle management.” Primary button: “Get started.” Account prompt and link: “Already have an account? Log in.” The explanatory text crosses the declared self-management claim boundary by implying therapeutic benefit and personalization without supporting outcome evidence. It should be removed or replaced before reuse. Because Figure 3 is a non-version-linked design composite, the wording documents a design-level claim-governance failure rather than proving display in version 1.0.5+14.

Panel B. Lifestyle questionnaire. Progress indicator: "3/20." Question: "How long is the interval between your last meal and your usual bedtime?" Response anchors: "Less than 1 hour," "1-2 hours," "2-3 hours," "3-4 hours," and "4 hours or more." Navigation buttons: "Next" and "Previous."

Panel C. GerdQ symptom-recording module. Title: “Today’s GerdQ.” Instruction: “Select the average frequency of each symptom during the past 7 days.” Six symptom rows are displayed, each with response boxes coded 0–3. Button: “Save.” The final Figure

3 removes the copyrighted Korean item wording because authorization for electronic use had not been confirmed for the fixed snapshot. The screen documents interface structure only; it does not establish diagnostic use or permission to deploy the questionnaire.

Panel D. Mission selection. Title: “Select missions.” Instruction: “Select up to three missions you need (3/3).” The source design composite showed the following example cards: “Avoid late-night meals or snacks within 3 hours before bedtime”; “Eat slowly and chew thoroughly”; “Take a light 30-minute walk after meals”; and “Drink 1.5–2 L of water per day.” Button: “Complete selection.” The final Figure 3 masks these card texts because they are non-versioned design examples, not the authoritative version 1.0.5+14 mission inventory. Supplementary Table S1 is the authoritative fixed and evidence-bounded content record.

Panel E. Daily mission check. Title: “Today’s missions.” The source design composite showed the date “Saturday, 17 May 2025” and repeated the first three example mission cards from Panel D. Status/action labels included “Completed” and “Mark complete.” Bottom navigation: “Home,” “Records,” “Missions,” “Report,” and “More.” The final Figure 3 masks the mission text to prevent inference that these examples were implemented in version 1.0.5+14.

Panel F. Weekly progress report. The source design composite showed the title “Weekly report,” the period “11–17 May 2025,” the metric label “Average GerdQ score,” a change label “Down 2 from the previous week,” daily values of 14, 13, 12, 11, 10, 12, and 9, a mission completion rate of 86% (18/21), and perceived symptom improvement of 70% rated moderate or higher. These values were invented for the interface mock-up and were not participant observations. The final Figure 3 masks the metric area and labels it as an illustrative mock-up so it cannot be mistaken for fixed-version functionality or study results.

GerdQ display boundary. The source report panel is a non-versioned design mock-up and is not evidence that version 1.0.5+14 displayed a GerdQ total or average to users. The fixed-version case record indicates that no total score, diagnostic threshold, or clinical interpretation was displayed. Any future score display would require confirmed licensing, validated scoring implementation, bounded interpretation, and clinical-safety review.

Figure-wide interpretation boundary. Figure 3 combines interface design materials that were not all linked to the fixed software release. Panel A is used to identify a design-level claim defect; Panel C documents structure with item wording removed; and Panels D–F show non-versioned layout concepts with content masked in the final figure. Dates, scores, percentages, and completion counts transcribed above are source mock-up values, not participant observations or study outcomes. The figure does not establish clinical effectiveness, safety, regulatory approval, or deployment status.

Supplementary Table S1. Sokcare lifestyle item–mission review and evidence-bounded revision.

The original mission column preserves the fixed content implemented in version 1.0.5+14 for auditability. Item-level review classified 18 of 20 mission statements as Revise and 2 as Retain; none was classified as Remove. Revised formulations specify content for a future version and were not deployed or clinically validated in the fixed prototype. The counts describe a content audit, not an effect estimate.

Response-code boundary. The fixed lifestyle questionnaire used item-specific wording and five ordered response codes (1-5). Table S1 reports the verified topic and mission text. The exact item-level anchor dictionary was not separately archived as a citable artifact in the materials used for this Perspective; therefore, unverified wording is not reconstructed here. Independent reproduction of the full input layer requires a version-controlled export of all Korean item texts, English translations, anchors, coding direction, and item-to-mission lookup before further public or clinical evaluation.

ID	Lifestyle topic	Original mission in version 1.0.5+14	Review decision	Evidence-bounded revision	Evidence context
1	Late evening meal	Eat before 19:00	Revise	Finish the last meal at least 3 h before planned recumbence when feasible.	Comparatively direct guidance [19,20,23]
2	Lying down within 2–3 h after a meal	Wait at least 2 h before lying down	Revise	Avoid lying down soon after meals and maintain an appropriate pre-recumbence interval, preferably around 3 h when feasible.	Comparatively direct guidance [19,20,23]
3	Rapid eating	Spend at least 15 min eating	Revise	Practice slower eating and adequate chewing without imposing a universal minimum meal duration.	Observational/conditional [52,53]
4	High-fat food	Avoid greasy food	Revise	Reduce foods repeatedly associated with personal symptoms rather than universally restricting high-fat foods.	Conditional and individualized [19,23,52]
5	Large meals or binge eating	Stop at approximately 80% fullness	Revise	Use smaller portions or divided meals when large meals are symptom-linked; avoid a fixed fullness threshold.	Observational/conditional [52–54]
6	Spicy or irritating food	Avoid irritating food	Revise	Modify only repeatedly identified personal food triggers.	Mixed evidence; individualized [19,23,52]
7	Frequent sweets	Limit sweets to once daily	Revise	Record intake and reduce excessive use without implying a specific GERD threshold.	Limited/indirect [54,55]
8	Caffeine intake	Avoid caffeine after 15:00	Revise	Adjust caffeine amount or timing when repeatedly associated with symptoms or sleep disruption.	Mixed evidence; individualized [19,23,52]
9	Insufficient sleep	Maintain at least 6 h sleep	Revise	Support regular sleep timing and adequate individualized sleep duration; no GERD-specific therapeutic threshold is assumed.	Conditional [19,20,54]
10	Poor sleep quality	Stretch for 30 min before bed	Revise	Offer a low-burden sleep-hygiene or relaxation routine without prescribing a fixed duration.	Indirect/conditional [19,24]
11	Weekly alcohol use	Avoid alcohol	Revise	Reduce or avoid alcohol when repeatedly associated with symptoms and consider amount and frequency.	Observational and dose-dependent [52,55]
12	Reflux after alcohol	Avoid food after alcohol and delay sleep	Revise	When alcohol is symptom-linked, reduce or avoid intake and maintain an appropriate meal–recumbence interval.	Individual symptom linkage [19,23]

ID	Lifestyle topic	Original mission in version 1.0.5+14	Review decision	Evidence-bounded revision	Evidence context
13	Current smoking	Reduce one cigarette daily	Revise	Encourage smoking cessation planning and use of professional cessation support.	Comparatively direct guidance [19,20,23,55]
14	High stress	Practice breathing twice	Revise	Offer a clearly specified diaphragmatic-breathing exercise as optional adjunctive support; avoid a fixed twice-daily dose or a general treatment claim.	Selected intervention evidence; heterogeneous protocols and populations [24–29]
15	Stress-related overeating	Pre-meal breathing and food diary	Retain	Retain as optional behavioral self-monitoring support; do not present it as GERD treatment.	Self-monitoring is a standard behavioral component described in BCTTv1 [43], which is a descriptive taxonomy and not efficacy evidence; direct GERD-specific evidence is limited [23,24]. Retained as low-risk optional support only.
16	Frequent anxiety or low mood	Use breathing content	Revise	Use breathing only as optional coping support and recommend professional evaluation for persistent or severe anxiety or low mood.	Association does not establish treatment [24,25]
17	Tight clothing	Choose loose clothing	Retain	Reduce postprandial abdominal pressure where relevant; present as optional low-risk behavior support.	Observational [53]
18	Recent weight gain	Perform ≥30 min aerobic activity	Revise	Link physical activity and weight-management support to baseline capacity and health status rather than a fixed duration.	Comparatively direct for overweight/obesity [19–23]
19	Low physical activity	Walk at least 5,000 steps	Revise	Use a graded activity target matched to baseline capacity; do not assume a universal 5,000-step therapeutic target.	Association evidence; target not clinically validated [56]
20	Prolonged sitting	Stretch hourly	Revise	Interrupt prolonged sitting with brief walking or stretching according to feasibility; GERD-specific benefit remains uncertain.	Indirect; physical-activity association evidence only [56]. BCTTv1 [43] is cited as descriptive labeling, not as efficacy evidence.

Version-control note. The evidence-bounded revisions in Table S1 are specifications for a future content release. They must not be represented as deployed in version 1.0.5+14 unless the revised application, content manifest, and change log are fixed under a new version identifier.

Supplementary Table S2. Source identification and selection summary

Source route	Records identified	Duplicates removed	Unique records/documents retrieved	Retained in final source set
PubMed/MEDLINE	1,248	51	1,197	7
WHO/UN/NIST/IMDRF/MFDS websites	9	0	9	9
Backward citation checking	Not prospectively enumerated	-	-	40
Total unique sources retained	-	-	-	56

Note. The three PubMed blocks retrieved 423, 769, and 56 records, respectively. After 51 duplicates were removed, 1,197 unique records remained; 7 were retained directly and 1,190 were not retained. Nine official documents and 40 sources from backward checking or targeted supplementation produced a final set of 56 sources. The retrieval count of the third block (56 records) and the size of the final source set (56 sources) coincide numerically but are unrelated quantities. Backward-search exclusions were not prospectively enumerated.

Supplementary Table S3A. Domain-level derivation audit trail

This domain-level table identifies the distinct blocking decision used to retain each gate or domain. Supplementary Table S3B maps every source in the final 56-source set to the framework or case element it informed.

Element	Distinct blocking decision	Primary source anchors	Reason retained separately
Gate 0	Is AI necessary and proportionate to the expected benefit?	WHO AI, NIST, Green AI, climate-impact review, NASSS [3–5,8,10,48]	Combines nonadoption as a legitimate outcome with an AI-specific necessity and proportionality decision that can retain rules, hold, or stop.
Domain 1	Is the intended purpose bounded and matched to valid evidence?	IMDRF SaMD, MFDS, DTx RWE, clinical guidance [11,13,14,19–23]	Clinical validity and claim boundaries require their own evidence package.
Domain 2	Can harms be escalated and can a human meaningfully intervene?	WHO AI, GMLP, DECIDE-AI, SPIRIT-AI, CONSORT-AI [3,4,7,45–47]	Clinical-safety ownership and escalation cannot be inferred from model performance.
Domain 3	Are data lawful, secure, representative, and traceable?	NIST, GMLP, IMDRF cybersecurity [5,7,12]	Data governance and cybersecurity require distinct controls and incident response.
Domain 4	Can outputs, versions, changes, and limitations be audited?	WHO AI, NIST, FUTURE-AI [3–6]	Traceability and explanation require documentation independent of clinical validity.
Domain 5	Who may be excluded, mismeasured, or differentially harmed?	WHO digital health/AI, FUTURE-AI, human-centered design [1,3,5,6,38–41]	Accessibility and bias need representative participation and subgroup evidence.
Domain 6	Can the service be maintained, integrated into care pathways, supported, and retired?	NASSS, CeHRes, DTx RWE [14,40,41,48]	Operational ownership, workflow integration, and lifecycle capacity can independently determine failure.
Domain 7	Is the intended use regulatorily, economically, and operationally viable?	MAST, IMDRF/MFDS, DTx RWE [11–15,49]	Regulatory evidence, implementation planning, reimbursement, and total lifecycle cost require distinct ownership.
Domain 8	Is computational and material complexity environmentally proportionate?	Green AI, sustainable health ethics, climate-impact review [8–10]	Environmental resource use is not captured adequately by clinical or economic value alone.

Supplementary Table S3B. Complete source-to-element mapping for the 56 retained sources

Reference numbers correspond to the main-manuscript reference list. G0 denotes Gate 0 and D1–D8 denote the eight readiness domains. Mappings identify the elements to which a source made a direct contribution in the present derivation or case-boundary interpretation; broad background relevance was not coded. A citation does not imply that the source validates the proposed framework.

Ref.	Source type	Mapped element(s)	Use in derivation or case interpretation
1	WHO digital-health strategy	D5, D6, D7	Equity, health-system integration, and organizational-resource context.
2	UN sustainable-development source	D5, D8	Equitable health and sustainability context.
3	WHO AI ethics guidance	G0, D2-D5, D7	Safety, autonomy, transparency, accountability, inclusion, and governance.
4	WHO AI regulatory guidance	G0, D1-D4, D7	AI regulatory lifecycle, evidence, risk, and governance expectations.
5	NIST AI risk framework	G0, D2-D6	Govern-Map-Measure-Manage functions and cross-cutting risk controls.
6	Healthcare AI consensus guide-line	D2, D4–D6	Direct guidance on safety, traceability, usability, fairness, and deployability across the health-AI lifecycle.
7	IMDRF AI/ML guidance	D1-D4, D6	Representative data, software engineering, human-AI teams, and monitoring.
8	Green-AI framework paper	G0, D8	Compute efficiency and transparent resource reporting.
9	Environmental-health AI ethics	D8	Environmental responsibility and justice in health-AI development.
10	Digital-health climate scoping review	G0, D8	System boundaries and material, energy, and carbon consequences.
11	IMDRF SaMD guidance	D1, D2, D7	Clinical evaluation and intended-use evidence requirements.
12	IMDRF cybersecurity guidance	D3, D6, D7	Total-product-lifecycle cybersecurity and incident response.
13	Korean MFDS DTx guidance	D1, D2, D7	Jurisdiction-specific authorization and review expectations.
14	DTx real-world-evidence framework	D1, D2, D6, D7	Direct guidance on clinical evidence, safety, implementation, deployment, and lifecycle monitoring for DTx.
15	Korean DTx narrative review	D7	Korean regulatory, market, and implementation context.
16	GERD clinical consensus	D1	Clinical definition and intended-condition boundary.
17	GERD pathophysiology review	D1, D2	Diagnosis and management rationale and safety boundaries.
18	GERD diagnostic consensus	D1, D2	Validated diagnostic context and limits of symptom-only interpretation.
19	ACG GERD guideline	D1, D2; Table S1	Clinical management, safety, and conservative lifestyle content.
20	ASGE GERD guideline	D1, D2; Table S1	Diagnosis, management, red flags, and evidence-bounded content.
21	AGA clinical-practice update	D1, D2; Table S1	Personalized management and claim boundaries.
22	Korean GERD guideline	D1, D2, D7; Table S1	Jurisdiction-relevant clinical evidence and treatment context.
23	GERD lifestyle review	D1; Table S1	Lifestyle recommendations and evidence-strength boundaries.
24	Psychosocial systematic review	D1; Table S1	Association evidence for sleep, anxiety, mood, and stress; no treatment inference.
25	Behavioral-therapy statements	D1, D2; Table S1	Behavioral support boundaries and need for appropriate clinical framing.
26	Breathing-exercise RCT	D1; Table S1	Direct but selected evidence for abdominal breathing.
27	Diaphragmatic-breathing RCT	D1; Table S1	Selected efficacy and mechanistic evidence.

Ref.	Source type	Mapped element(s)	Use in derivation or case interpretation
28	Breathing systematic review	D1; Table S1	Synthesis of heterogeneous breathing-exercise evidence.
29	Breathing review/meta-analysis	D1, D2; Table S1	Efficacy and safety evidence with bounded interpretation.
30	GERD-app user-centered-design study	D5, D6	User-centered requirements and development-process evidence.
31	GERD-app systematic review	D1, D2, D6, D7	App quality, regulation, and professional-involvement gaps.
32	GERD-app quality assessment	D1, D2, D5, D6	Content quality, safety, and usability limitations.
33	GI-app store evaluation	D1, D2, D5-D7	Cross-app quality, accessibility, and implementation concerns.
34	GERD mobile-health RCT	D1, D6	Example of prospective clinical and implementation evaluation.
35	GERD electronic-diary cohort	D1, D4, D6	Patient-reported outcome capture and digital symptom monitoring.
36	mHealth sustainability evaluation	D5-D8	Multidimensional social, organizational, economic, and environmental assessment.
37	Human-centered mHealth development	D5, D6, D8	Participatory design and sustainable app development.
38	CeHRes framework	D5-D7	Iterative development, implementation, evaluation, and contextual fit.
39	CeHRes Roadmap 2.0	D5-D7	Updated human-centered and implementation-lifecycle process.
40	IDEAS framework	D1, D5, D6	Behavior-change intervention design, assessment, and dissemination.
41	mERA reporting checklist	D4, D6	Transparent reporting of mobile-health implementation.
42	TIDieR checklist	D1, D4	Replicable intervention and content specification.
43	BCT Taxonomy v1	D1, D4; Table S1	Behavior-change content labeling; used for description, not efficacy claims.
44	MRC complex-intervention guidance	D1, D5, D6	Context-sensitive development and staged evaluation.
45	DECIDE-AI reporting guideline	D2, D4, D6	Early clinical evaluation and human-AI interaction reporting.
46	CONSORT-AI extension	D1, D2, D4	AI clinical-trial reporting requirements.
47	SPIRIT-AI extension	D1, D2, D4	AI clinical-trial protocol and safety specification.
48	GERD lifestyle systematic review	D1; Table S1	Evidence for dietary and lifestyle associations; supports conservative wording.
49	GERD lifestyle multicenter survey	D1; Table S1	Observational lifestyle associations and individualized interpretation.
50	Korean lifestyle cross-sectional study	D1, D5; Table S1	Korean dietary/lifestyle associations and population context.
51	GERD population study	D1, D5; Table S1	Dose- and context-dependent risk-factor associations.
52	Physical-activity review/meta-analysis	D1; Table S1	Activity association; no universal step or duration threshold.
53	NASSS framework	G0, D5-D7	Legitimate nonadoption, scale-up, spread, and sustainability.
54	MAST framework	D1, D5-D8	Multidimensional telemedicine assessment and transferability.
55	GerdQ development study	D1, D4	Original diagnostic purpose, structure, scoring, and interpretation boundary.
56	Korean GerdQ validation	D1, D5	Korean-language validation and population-specific use boundary.

Supplementary Table S4. Basis and interpretation boundary for the comparative positioning in Table 2

The table records the source scope used to position each framework. It does not assess quality, completeness, or superiority.

Framework or guidance	Source scope used in the comparison	Interpretation boundary
WHO digital health and AI guidance [1,3,4]	Digital health is framed as a health-system strategy, while the AI guidance provides ethical and regulatory lifecycle principles.	Treated as a guidance family rather than one unified framework; it does not itself provide an AI-versus-rules decision algorithm.
NIST AI RMF [5]	Voluntary cross-sector risk management for the design, development, use, and evaluation of AI systems, organized as Govern, Map, Measure, and Manage.	Health-specific clinical ownership, claim boundaries, and red-flag symptom detection and escalation pathway require additional specification.
FUTURE-AI [6]	Consensus guidance for the development and deployment of trustworthy and deployable AI tools in healthcare.	Applies to AI systems under development or deployment; it is not framed as a decision about whether to add AI to a non-AI platform.
NASSS [48]	A framework for adoption, nonadoption, abandonment, scale-up, spread, and sustainability of health and care technologies.	Provides a strong basis for legitimate nonadoption, but not an AI-specific fixed-rule comparator or data-readiness test.
MAST [49]	A structure combining preceding considerations, multidimensional assessment, and transferability assessment for telemedicine.	Not designed for AI-specific model and data governance or a pre-development rule-versus-AI decision.
CeHRes Roadmap 2.0 [40,41]	A human-centered roadmap in which eHealth development, implementation, and evaluation are ongoing, intertwined, and iterative.	Not an AI-specific necessity gate and does not prescribe bounded AI adoption decisions.
DTx RWE Framework [14]	A lifecycle approach to evidence-based DTx design, development, testing, deployment, and monitoring.	Does not specifically test whether AI should be added to a functioning rule-based platform before model development.

Supplementary Table S5. Reusable qualitative assessment form

Assessment identification (complete before rating). Platform and version: __________ Proposed AI function: __________ Intended use, population, and setting: __________ Rule/non-AI comparator: __________ Regulatory context: __________ Date: __________ Assessors, roles, and conflicts: __________

For each element, cite versioned evidence identifiers and record Met (M), Partially met (PM), Unmet (U), or Not applicable (N/A). N/A requires a written risk-based justification. Unsupported verbal assurance is not admissible evidence. Do not sum statuses across rows.

Element	Observable minimum criteria	Required evidence to cite	Assessment record
Gate 0	The proposed AI decision and failure mode are bounded; the non-AI comparator is fixed and clinically justified; incremental benefit, acceptable harm, equity constraints, organizational burden, cost, and environmental/resource limits are prespecified; data and lifecycle governance are feasible.	AI use-case specification; versioned comparator; baseline technical/clinical evidence; outcomes and minimum meaningful difference; harm/equity constraints; data-feasibility and lifecycle-burden assessment.	Status: ___<br>Evidence IDs: ___<br>Decision: ___
D1	Intended purpose, claims, population, setting, and changed decision are bounded; content and outcomes are evidence-linked; labels/outcomes are valid for the proposed use.	Intended-use statement; content-evidence map; claim-evidence matrix; prespecified patient-relevant outcomes; clinical review; prospective evidence plan/results.	Status: ___<br>Evidence IDs: ___<br>Owner/date: ___
D2	Red flags, contraindications, medication limits, escalation, adverse-event handling, monitoring limits, and meaningful human authority are defined and tested for the intended risk.	Safety requirements; hazard/risk analysis; red-flag and referral pathway; oversight and override roles; incident/adverse-event plan; human-factors test records.	Status: ___<br>Evidence IDs: ___<br>Owner/date: ___
D3	Data use is lawful, necessary, traceable, representative, secure, retained/deleted appropriately, and covered by incident response throughout the lifecycle.	Data inventory and provenance; lawful-basis/consent record; access and retention policy; security and vulnerability tests; breach response; AI data-lineage and missingness plan where applicable.	Status: ___<br>Evidence IDs: ___<br>Owner/date: ___
D4	Users and reviewers can identify active versions, inputs, outputs, limits, changes, and accountable decisions; explanations support action at the intended risk level.	Rule/model card; versioned specifications; change log; decision log; limitation and uncertainty statements; audit trail; content/model approval record.	Status: ___<br>Evidence IDs: ___<br>Owner/date: ___
D5	Relevant users can access and use the service; representation, measurement error, differential performance, and downstream harm are examined for prespecified groups.	Accessibility audit; participatory/user testing; subgroup and representativeness plan; differential-performance/harm analysis; mitigation and monitoring plan.	Status: ___<br>Evidence IDs: ___<br>Owner/date: ___
D6	Architecture, testing, dependencies, maintenance, monitoring, workflow, staffing, support, rollback, and retirement are owned and feasible in the intended organization.	Architecture/test plan; dependency inventory; maintenance and monitoring plan; clinical/operational workflow; staffing and support model; rollback and retirement procedure.	Status: ___<br>Evidence IDs: ___<br>Owner/date: ___
D7	Intended use, regulatory classification, quality/evidence strategy, workflow integration, reimbursement/service model, procurement, and total lifecycle cost are viable.	Regulatory and QMS strategy; clinical-evaluation plan; implementation/service model; health-economic analysis; procurement/reimbursement pathway; lifecycle budget.	Status: ___<br>Evidence IDs: ___<br>Owner/date: ___
D8	The rule-versus-AI comparison uses the same task/workload and a stated system boundary; computational/material effects are measured or transparently proxied; simplification and retirement conditions are defined.	Task-matched benchmark; model/API and hardware/hosting inventory; inference, storage, and transfer workload; latency and energy measurement/proxy; carbon assumptions; sensitivity analysis; retirement criteria.	Status: ___<br>Evidence IDs: ___<br>Owner/date: ___

Status and decision rules. M means the required evidence is complete, versioned, attributable to an accountable owner, and independently checked when risk warrants. PM means relevant evidence exists but is incomplete or not independently verified. U means required evidence or governance is absent. N/A requires explicit intended-use and risk justification. Retain rules when clinically or operationally meaningful incremental AI benefit has not been prespecified over a valid non-AI baseline. Hold when a potentially remediable evidence gap or any

unmet blocking condition remains. Stop when the proposed AI function creates an unacceptable or unmanageable safety, legal, equity, organizational, economic, or environmental burden. Proceed only to the next defined evidence-generation stage when Gate 0 is passed and no blocking condition remains; proceed does not itself authorize deployment or a clinical claim.

No aggregate score. A favorable finding in one domain cannot compensate for an unmet blocking condition in another. The completed form should retain the cited evidence identifiers, assessor names and expertise, conflicts of interest, disagreements, adjudication, final decision, owner, and review date.

Supplementary Table S6. Sokcare case-evidence register (artifact keys A1-A4)

The register defines what each artifact key can support in the author-conducted worked example. All classifications remain author-reported and provisional. Absence of a public artifact, independent audit, or exact version linkage is recorded as a limitation rather than treated as evidence of readiness.

Key	Versioned artifact	Scope examined	Availability and evidentiary limitation	Supports
A1	Fixed Flutter source snapshot, version 1.0.5+14	Application structure; onboarding; lifestyle and symptom modules; candidate, reminder, completion, and reporting logic; local state.	Proprietary and security-sensitive; no repository path, commit hash, independent code audit, or public archive was provided. Supports only author-reported implementation statements, not clinical validity or security assurance.	G0; D1-D4; D6-D8
A2	Item-mission content map and deterministic rule specification	Twenty topic-mission records in Table S1; evidence-bounded revision status; Methods S2, Algorithm S1, and Decision Table S1.	Included in this supplement at topic/mission and algorithm level. Exact item wording and anchor dictionary were not separately archived; executable code is not public. Supports traceability, not clinical effectiveness.	G0; D1; D4; D7
A3	Prototype interface screens in Figure 3 and Supplementary Text S1	Visible onboarding, questionnaire, GerdQ structure, mission selection/check, and weekly-report mock-up.	Values are illustrative and not participant outcomes. Full GerdQ wording is omitted pending authorization. Screens do not prove deployment in the fixed snapshot unless version linkage is independently verified.	D1; D2; D5
A4	Backend and data-flow documentation in Figure 2 and case narrative	SharedPreferences local state; persistent pseudonymous UUID; Supabase access, survey-response, and mission-submission events; web administration.	Author-reported architecture; operational configuration is not public. No independent access-control, row-level security, vulnerability, penetration, retention/deletion, or incident-response audit was provided.	D3; D6; D8

Minimum future evidence-package requirement. Before any renewed public or clinical evaluation, the authors should preserve a checksum- or commit-linked source snapshot, content manifest, complete item/anchor dictionary, change log, safety requirements, data inventory, security-test record, accessibility/usability evidence, and named approval owners under one release identifier. That future package would replace broad author-reported artifact keys with independently inspectable records.
